# Distinct Signaling Pathways Regulate TREM2 Phagocytic and NFκB Antagonistic Activities

**DOI:** 10.3389/fncel.2019.00457

**Published:** 2019-10-10

**Authors:** Hailan Yao, Kyle Coppola, Jonas Elias Schweig, Fiona Crawford, Michael Mullan, Daniel Paris

**Affiliations:** ^1^The Roskamp Institute, Sarasota, FL, United States; ^2^James A. Haley Veterans’ Hospital, Tampa, FL, United States

**Keywords:** Alzheimer’s disease, TREM2, inflammation, signaling pathways, phagocytosis, SYK, DAP12, APOE

## Abstract

Several genetic variants of the Triggering Receptor Expressed on Myeloid Cells-2 (TREM2) have been shown to increase the risk of developing Alzheimer’s disease (AD) supporting a role of microglia and immune cells in the pathobiology of AD. We have employed an ectopic model of TREM2 and DAP12 expression in HEK293 cells to study selectively TREM2 dependent signaling and phagocytic functions and evaluated the effects of some of the TREM2 mutations associated with AD. We show that shedding of the TREM2 N-terminal domain does not affect the inhibition of NFκB activation induced by TREM2 while it completely blocks phagocytosis suggesting that TREM2 anti-inflammatory properties can be mediated by the TREM2 C-terminal fragment while the phagocytic activity requires the full-length receptor. In addition, we confirm in that model that apolipoprotein E (APOE) is a ligand for TREM2 and triggers TREM2 signaling. In particular, we show that APOE4 stimulates spleen tyrosine kinase (SYK) activation more potently than APOE2 in a TREM2 dependent manner. Interestingly, TREM2 appears to antagonize NFκB activation induced by phorbol ester but is unable to prevent TNFα induction of NFκB activation suggesting that TREM2 antagonizes inflammatory events triggered downstream of PKC. TREM2 mutations drastically impact TREM2 phagocytosis as well as its ability to antagonize NFκB activation and notably prevent the activation of the PI3K/AKT pathway observed with wild-type TREM2. Overall our data suggest that TREM2 dependent phagocytosis requires an activation of the SYK/PI3K/AKT/PLCγ pathways while the suppression of NFκB activation by TREM2 is independent of SYK, PI3K, and PLCγ activities. This model of ectopic TREM2-DAP12 co-expression appears suitable to study TREM2 signaling as several biological functions of TREM2 and TREM2 mutations that have been previously described in myeloid and microglial cells were also replicated in this model.

## Introduction

The brain of Alzheimer’s disease (AD) patients is characterized by the presence of three pathologies that are central to the AD process: extracellular accumulation of cerebral β-amyloid (Aβ); intraneuronal accumulation of hyperphosphorylated and aggregated tau, and; inflammation (Hardy and Selkoe, 2002; De Strooper and Karran, 2016; Hammond et al., 2019). Much evidence, from many groups, suggests that these three AD pathologies interact synergistically each exacerbating the others resulting in synaptic and neuronal loss (Yoshiyama et al., 2007; Gorlovoy et al., 2009; Roe et al., 2011).

Genetic studies have identified variants in the gene TREM2 (triggering receptor expressed on myeloid cells 2) that confer increased risk for developing AD (Guerreiro et al., 2013; Jonsson et al., 2013), directly implicating immunoinflammatory responses in AD pathobiology as TREM2 encodes a receptor that is exclusively expressed on immune cells within the CNS including infiltrating monocytes/macrophages and microglia (Colonna, 2003; Hickman et al., 2013; Fahrenhold et al., 2018). Although, TREM2 mutations are rare, their observed effect size is comparable to the *APOE*ε*4* allele, which represents the strongest genetic risk factor for late onset AD (Ulrich et al., 2017; Yeh et al., 2017; Carmona et al., 2018). The most common AD TREM2 variant results from a single nucleotide polymorphism encoding an arginine to histidine missense substitution at the amino acid 47 (R47H) (Guerreiro et al., 2013; Jonsson et al., 2013).

Interestingly, APOE is a known ligand for TREM2 and several AD-associated mutations in TREM2 impair APOE binding (Atagi et al., 2015; Yeh et al., 2016) suggesting that these two AD risk genes could be mechanistically linked. Aβ oligomers have also been shown to interact with TREM2 with high affinity and to induce NFAT (nuclear factor of activated T cell) signaling while in TREM2 AD variants, although the Aβ affinity for TREM2 remains unchanged, NFAT signaling induced by Aβ oligomers is reduced suggesting a partial loss of TREM2 function (Lessard et al., 2018). TREM2 is involved in microglia phagocytosis and activation as TREM2 knockdown inhibits phagocytosis and stimulates the production of inflammatory cytokines by microglia while TREM2 overexpression has the opposite effect (Takahashi et al., 2005). BV2 microglial cells expressing AD TREM2 variants show impaired phagocytic activity suggesting TREM2 loss of function for these variants (Kleinberger et al., 2014).

TREM2 signals through its association with TYRO protein tyrosine kinase binding protein (TYROBP), also known as DNAX-activating protein of 12 kDa (DAP12), which recruits the spleen tyrosine kinase (SYK) through its cytosolic immunoreceptor tyrosine-based activation motifs (ITAMs) (Peng et al., 2010). SYK has been therefore postulated to be a key kinase required to transduce TREM2 signaling pathways. TREM2 signaling has anti-inflammatory consequences and has been shown to antagonize Toll-like receptor (TLR-4) mediated inflammation by modulating the JNK and NFκB signaling pathways (Takahashi et al., 2005; Hamerman et al., 2006; Zhong et al., 2017a). Most of the studies on TREM2 signaling have used anti-TREM2 antibodies to stimulate the TREM2 receptor promoting the interaction between TREM2 and DAP12 and the recruitment of SYK (Varnum et al., 2017). So far, the only attempts to study TREM2 signaling in response to receptor ligation with APOE have been conducted by using indirect calcium-driven reporter systems without clearly delineating which signaling pathways are triggered or antagonized upstream of the reporter. Such functional analyses have however, suggested that APOE is an agonist of TREM2 (Jendresen et al., 2017) and that several TREM2 variants associated with AD impair TREM2 activation whereas other variants opposingly increase TREM2 activation in response to phosphatidylcholine and other lipid ligands (Song et al., 2017) suggesting that AD TREM2 mutations are not simply loss of function mutations as previously thought.

To investigate the functional role of TREM2 on AD pathology, several studies have tested the impact of TREM2 deficiency on Aβ accumulation and tau pathology using various transgenic mouse models of AD as AD associated TREM2 mutations were assumed to result from TREM2 loss-of-function phenotype (Cheng-Hathaway et al., 2018; Song et al., 2018). The studies conducted using models of Aβ accumulation gave puzzling results and suggest that TREM2 deficiency reduces the Aβ pathology early on Jay et al. (2015, 2017) but increases Aβ deposition in older mice (Wang et al., 2016; Jay et al., 2017). Other studies show opposite data with increased Aβ deposition in younger TREM2 deficient mice and no change in Aβ deposition in older animals (Parhizkar et al., 2019). Similarly, the impact of TREM2 deficiency on tau pathology is unclear with studies suggesting that TREM2 deficiency exacerbates the tau pathology (Bemiller et al., 2017) or on the opposite prevents the neurodegeneration induced by the tauopathy (Leyns et al., 2017). In addition, differential effects of partial and complete loss of TREM2 have been observed in a mouse model of tauopathy showing protective effects of complete TREM2 deficiency but exacerbation of the tau pathology in TREM2 haploinsufficient mice (Sayed et al., 2018). Therefore, it is not settled whether TREM2 plays a beneficial or detrimental role in AD and these studies do not shed light on the exact molecular mechanisms that link TREM2 variants to the AD pathobiology.

TREM2 deficiency consistently leads to a reduction in myeloid cell accumulation around Aβ deposits supporting a possible role of TREM2 in myeloid cell survival, proliferation, and chemotaxis (Jay et al., 2015, 2017; Wang et al., 2016; Yuan et al., 2016). It has been suggested that TREM2-dependent microglial functions limit amyloid plaques growth early by promoting Aβ phagocytosis but not late during the disease by increasing the levels of APOE around amyloid deposits, hence promoting further Aβ aggregation (Parhizkar et al., 2019) given the known role of APOE on Aβ deposition. AD TREM2 mutants may therefore enhance Aβ deposition during the early stage of AD while leading to a reduction in APOE production by microglia and decreased APOE levels in senile plaques suggesting that TREM2 may have protective functions early during amyloidogenesis (Parhizkar et al., 2019).

As indicated earlier, DAP12 is a signaling adapter protein that pairs with TREM2 and is required to mediate TREM2 signaling. Interestingly, DAP12 deletion has been shown to improve learning behavior and synaptic function in a mouse model of tauopathy (Audrain et al., 2018) and to reverse the behavioral, electrophysiological alterations and neuro-inflammation in a mouse model of β-amyloidosis (Haure-Mirande et al., 2019). It remains possible that aberrant TREM2 signaling induced by TREM2 mutations plays a key role in the development of AD suggesting that the study of TREM2 signaling may lead to the discovery of therapeutic targets downstream of TREM2 that could be manipulated to reverse the effects of TREM2 mutations.

TREM2 is a negative regulator of TLR4 mediated inflammation (Takahashi et al., 2005; Hamerman et al., 2006; Zhong et al., 2017a; Li and Zhang, 2018) but the exact molecular mechanisms responsible for this effect are not fully understood. In addition, the impact of TREM2/DAP12 on inflammation mediated by other inflammatory stimuli beside LPS (TLR-4 agonist) remains to be examined.

The study of TREM2 signaling in immune cells or microglia is particularly challenging because these cells express many receptors, besides TREM2, that are also signaling by engaging DAP12. Therefore, it is particularly complex to delineate specific TREM2 signaling pathways from pathways influenced by other receptors recruiting DAP12 in these cells. In addition, immune cells and microglia express multiple phagocytic receptors which complicates the study of TREM2-dependent phagocytic activity and the signaling mechanisms involved in this process. To overcome these difficulties and allow to assess selectively TREM2 dependent phagocytic and anti-inflammatory functions, we elected to express TREM2 and DAP12 in HEK293 cells that do not express DAP12 or TREM2 and are non-phagocytic. Several groups have used similar TREM2 and DAP12 co-expression in HEK293 cells to study TREM2 biology including TREM2 signal transduction and function as well as TREM2 shedding (Kleinberger et al., 2014; Feuerbach et al., 2017; Thornton et al., 2017). In this study, we investigated the impact of TREM2/DAP12 on PMA (phorbol-12-myristate-13-acetate) and TNFα induction of NFκB and assessed the impact of TREM2 processing on inflammation and phagocytosis. In addition, we determined whether SYK stimulation by TREM2/DAP12 was required to mediate TREM2 dependent phagocytic and anti-inflammatory activities. Our data differentiate TREM2 signaling events responsible for its anti-inflammatory and phagocytic activities and reveal the impact of the AD associated TREM2 R47H variant on these events. Overall our data show that TREM2/DAP12 anti-inflammatory activity is mediated by signaling events distinct from those required to mediate TREM2 dependent phagocytic activity. In addition, we show that TREM2 processing has no influence on TREM2 anti-inflammatory activity but greatly impacts TREM2 dependent phagocytosis.

## Materials and Methods

### Reagents

12-*O*-Tetradecanoylphorbol 13-acetate (PMA), human recombinant TNF-α, dimethyl sulfoxide (DMSO), 2-mercaptoethanol, sodium chloride, phenylmethylsulfonyl fluoride (PMSF), TAPI-1, DAPT, and β-secretase inhibitor IV (C3) were purchased from Sigma-Aldrich (St. Louis, MO, United States). APOE2 and APOE4 were obtained from Biovision (Milpitas, CA, United States). Go 6983, Ro 32-0432, Wortmannin and LY294002 were purchased from Tocris (Minneapolis, MN, United States). All antibiotics, fungizone, PBS, culture media, and fetal bovine serum were purchased from Invitrogen (Carlsbad, CA, United States). The MPER reagent and the cocktail of protease/phosphatase inhibitors were purchased from Thermo Fisher Scientific (Waltham, MA, United States).

### DNA Constructs

A muliticistronic vector with “self-cleaving” 2A peptide sequence (pcDNA 3.1-P2A, GenScript) has been used to co-express human TREM2 and DAP12 genes from a single mRNA. Human TREM2 and DAP12 cDNA sequences were amplified by PCR, digested with restriction enzymes and sequentially inserted into the pcDNA3.1-P2A vector to develop the pcDNA3.1-TREM2-P2A-DAP12 plasmid. TREM2 mutants (Y38C and R47H) were generated in the pcDNA3.1-TREM2-P2A-DAP12 construct by using the Q5 site-directed mutagenesis kit (New England BioLabs), according to the manufacturer’s instructions. All constructs were confirmed by DNA sequencing. SYK shRNAs(E9) and a scrambled control shRNA (NS) were cloned into the GIPZ lentiviral vector and were purchased from Origene (Rockville, MD, United States).

### Cell Culture and Transfection

HEK293 cells were purchased from American Type Culture Collection (ATCC, Manassas, VA, United States) and maintained in DMEM medium supplemented with 10% fetal bovine serum, GlutaMAX, and 1% penicillin/streptomycin/fungizone (Thermo Fisher Scientific, Waltham, MA, United States). For stable transfection, HEK293 cells and a stable NFκB luciferase reporter cell line of HEK293 (Panomics, Fremont, CA, United States) were grown on 6-well plates until reaching 70–80% confluence and transfected using lipofectamine 2000 (Invitrogen, Carlsbad, CA, United States). After 48 h, transfected cells were placed into fresh medium in the presence of 500 μg/ml G418 (Invitrogen, Carlsbad, CA, United States) or 500 μg/ml G418 plus 100 μg/ml hygromycin B for the NFκB luciferase cells for selection. After 14 days in culture, resistant cells were trypsinized and expanded.

### Immunoblotting

HEK293 cells were lysed in mammalian protein extraction reagent (MPER, Thermo Fisher Scientific, Waltham, MA, United States) containing Halt protease and phosphatase single use inhibitor (Thermo Fisher Scientific, Waltham, MA, United States). Lysates were separated by SDS-PAGE using Criterion TGX gels (Bio-Rad, Berkeley, CA, United States), and transferred onto 0.2 μM PVDF membranes (Bio-Rad, Berkeley, CA, United States). Membrane were blocked with 5% non-fat milk in TBS for 1 h, incubated with anti-TREM2, anti-DAP12, anti-Phospho-Akt (Ser473), anti-AKT, anti-IκBα, anti- Phospho-NF-κB p65 (Ser536), anti-NF-κB p65, anti-Phospho-MARCKS (Ser167/170), anti-MARCKS, anti-Actin (1:1000, Cell Signaling, Danvers, MA, United States), anti-Phospho-Syk (Tyr525/526) (1:1000, Abgent, United States), or anti-SYK (4D10, 1:1000, Santa Cruz, CA, United States) primary antibody overnight at 4°C, and incubated in HRP-conjugated anti-Rabbit or anti-Mouse secondary antibody (1:3000, Cell Signaling, Danvers, MA, United States) at room temperature for 1 h. Western blots were visualized using SuperSignal West Femto Maximum Sensitivity Substrate (Thermo Fisher Scientific, Waltham, MA, United States). Signals were quantified using ChemiDoc XRS (Bio-Rad, Berkeley, CA, United States) and densitometric analysis were performed using Image Lab (Bio-Rad, Berkeley, CA, United States).

### Cell Immunostaining

HEK293 cells, grown on poly-D-lysine coated coverslips, were washed with PBS and fixed in 4% formaldehyde in PBS for 10 min. Cells were then washed three times in PBS, permeabilized and blocked using 5% BSA,0.3% Triton X-100 in PBS at room temperature for 1 h. Cells were then incubated with anti-TREM2 (1:100, R&D, United States), anti-DAP12 (1:100, Santa Cruz, CA, United States), and anti- Phospho-Akt (Ser473) (1:100, Cell Signaling, Danvers, MA, United States) primary antibodies overnight at 4°C. Cells were then washed three time and anti-goat secondary Alexa Fluor 488 conjugate, anti-mouse secondary Alexa Fluor 555, and anti-rabbit secondary Alexa Fluor 647 (1:1000, Invitrogen, United States) antibodies were added at room temperature for 1 h. Cells were washed three times and then mounted in Fluoroshield with DAPI (Sigma-Aldrich, St. Louis, MO, United States). Confocal microscopy was carried out using the LSM800 Laser Confocal Scanning Microscope (Carl Zeiss Microscopy, Thornwood, NY, United States), the ZEN Blue 2.1 software (Carl Zeiss AG, Germany) and a 63 × objective.

### Phagocytosis Assays

All cells were plated at a density of 100,000 cells in 96-well cell culture plates and cultured for 24 h. pHrodo *Escherichia coli* bioparticles were dissolved in live cell imaging buffer (Invitrogen, Carlsbad, CA, United States) at a concentration of 1 μg/ml and incubated at 37°C with the different cell lines in a Biotek Synergy HT reader. The uptake of *E. coli* bioparticles was measured by quantifying fluorescent emission (Ex 560 nm/Em 585 nm) every 10 min for 2 h. Phagocytosis was quantified by measuring the average amount of bioparticles uptake in mFU/min during the 2 h incubation period.

### NFκB Luciferase Activity Measurements

NFκB activation was triggered with 200 nM PMA or 10 μg/ml TNFα for 4 h, and NFκB luciferase activity was quantified by chemiluminescence using the Luc-Screen Extended Glow kit (Applied Biosystems, Foster City, CA, United States) with a Synergy HT chemiluminescence reader (Biotek) as we previously described (Paris et al., 2011).

### Statistical Analysis

Data were analyzed and plotted with GraphPad Prism (GraphPad Software, Inc., San Diego, CA, United States). The Shapiro–Wilk test for normality was used to test for Gaussian distribution. Statistical significance was determined by either one-way ANOVA (for comparisons of three or more groups), two-way ANOVA followed by *post hoc* analysis using Bonferroni’s correction or *t*-test where appropriate. All data are presented as mean ± standard deviation (SD) and *p* < 0.05 was considered significant (^∗^*p* < 0.05, ^∗∗^*p* < 0.01, ^∗∗∗^*p* < 0.001, ^∗∗∗∗^*p* < 0.0001).

## Results

### Generation of HEK293 Stable Cell Lines Co-expressing TREM2 and DAP12

TREM2 is a phagocytic receptor exclusively expressed in myeloid cells which signals trough the adaptor protein DAP12 following the binding of ligands to TREM2. It has been shown that some TREM2 ligands induce the phosphorylation of two tyrosine residues within the ITAM domain of DAP12 resulting in the recruitment and activation of SYK and downstream signaling molecules including PI3K and PLCγ (Takahashi et al., 2005; Otero et al., 2009; Peng et al., 2010; Wang et al., 2015; Colonna and Wang, 2016). In microglia and myeloid cells, DAP12 also form complexes with multiple receptors beside TREM2 which complicates the study of TREM2 signaling as signals attributed to TREM2 may emerge from other receptors that associate with DAP12. In addition, multiple phagocytic receptors are also expressed by myeloid cells which render difficult the study of specific TREM2 phagocytic functions. To overcome these difficulties and examine more selectively the role of TREM2 signaling in inflammation and phagocytosis, we induced an ectopic expression of TREM2 and DAP12 in non-immune and non-phagocytic HEK293 cells. We also selected HEK293 cells because they express SYK, PI3K, and PLCγ which are signaling molecules known to be recruited following TREM2 ligation in myeloid cells (Kleinberger et al., 2014; Licona-Limon et al., 2015).

**FIGURE 1:**
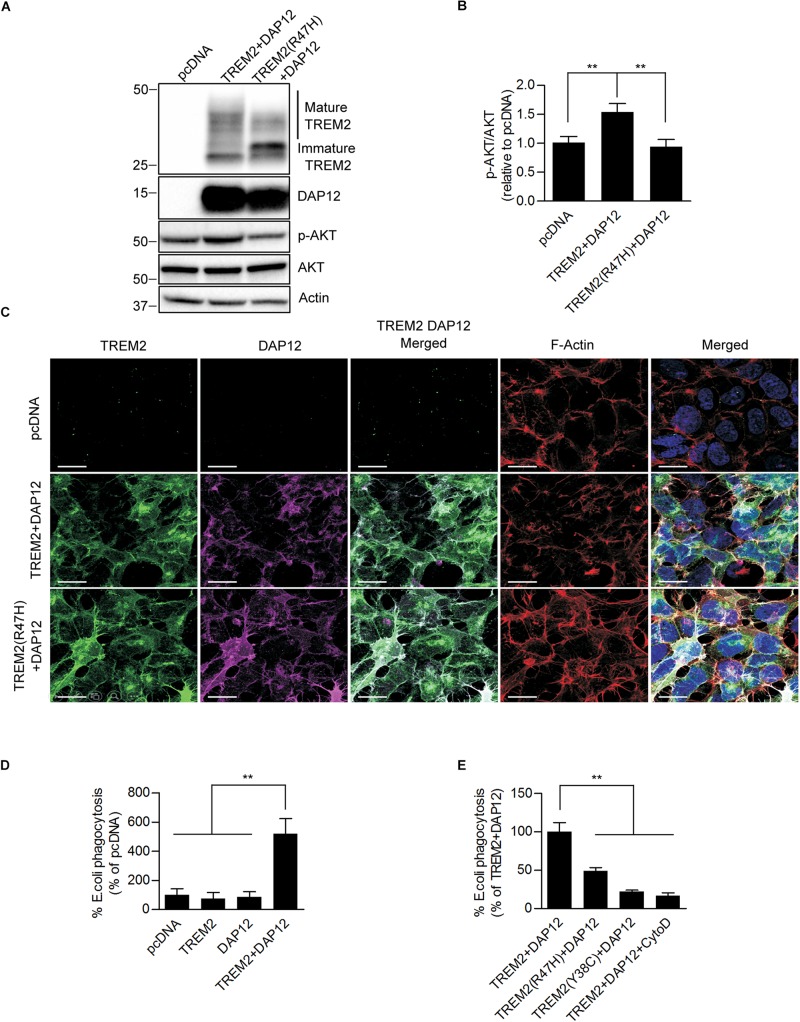
Ectopic expression of TREM2 and DAP12 induces AKT activation and phagocytosis in HEK293 cells. **(A)** Western-blot analysis of lysates from HEK293 cells transfected with pcDNA (control empty vector), TREM2 + DAP12 and TREM2 (R47H) + DAP12 constructs. A representative western-blot showing the impact of TREM2 + DAP12 and TREM2(R47H) + DAP12 expression on p-AKT(Ser473) compared to HEK293 cells transfected with the empty pcDNA vector (control) is shown. **(B)** Histogram representing the quantification by western-blots of p-AKT(Ser473) normalized to total AKT in HEK293 cells transfected with pcDNA (empty vector), TREM2 + DAP12 and TREM2(R47H) + DAP12 constructs. Data represent the average ± SD p-AKT/Total AKT levels standardized to values obtained in HEK293 cells transfected with the pcDNA empty vector; (*n* = 4) for each experimental condition; statistical significance (^∗∗^*p* < 0.01) was evaluated by ANOVA followed by *post hoc* analysis using Bonferroni corrections. **(C)** Representative laser confocal images of HEK293 cells transfected with pcDNA (empty vector), TREM2 + DAP12 and TREM2(R47H) + DAP12 following immunostaining with N-terminal TREM2 (green), DAP12 (purple) antibodies. Cells were counterstained with DAPI (blue) and fluorescent phalloidin (red) to stain F-actin. TREM2 and DAP12 expression was detected in HEK293 cells transfected with TREM2 + DAP12 and TREM2(R47H) + DAP12 constructs while no expression of TREM2 and DAP12 was observed in HEK293 cells transfected with the empty vector (pcDNA). Colocalization between TREM2 and DAP12 was observed both in cells co-expressing wild-type TREM2 and DAP12 and in cell co-expressing the TREM2 R47H mutation and DAP12. The white scale bar represents 20 μm. **(D)** Phagocytosis of pHrodo *E. coli* bioparticles conjugate in pcDNA, TREM2, DAP12, or TREM2 + DAP12 transfected HEK293 cells. Data are represented as mean ± SD from at least two independent cell culture experiments and expressed relative to data obtained in HEK293 transfected with the pcDNA empty vector; (*n* = 6) for each experimental condition; statistical significance (^∗∗^*p* < 0.01) was evaluated by ANOVA followed by *post hoc* analysis using Bonferroni corrections. Data show that the co-expression of DAP12 with TREM2 significantly stimulates the phagocytosis of pHrodo *E. coli* bioparticles whereas DAP12 or TREM2 expression alone are not sufficient to promote phagocytic activity compared to HEK293 transfected with the empty vector pcDNA (control). **(E)** Phagocytosis of pHrodo *E. coli* bioparticles conjugate in TREM2 + DAP12, TREM2(R47H) + DAP12, and TREM2(Y38C) + DAP12 transfected HEK293 cells. TREM2 + DAP12 transfected HEK293 cells were also treated with cytochalasin D (10 μM), an inhibitor of actin polymerization which blocks phagocytosis (positive control). Data are represented as means ± SD from at least two independent cell culture experiments and expressed as a percentage of the amount of phagocytosis quantified in TREM2 + DAP12 transfected HEK293 cells; (*n* = 6) for each experimental condition; statistical significance (^∗∗^*p* < 0.01) was evaluated by ANOVA followed by *post hoc* analysis using Bonferroni corrections. Data show that that cytochalasin D antagonizes TREM2 + DAP12 dependent phagocytic activity while TREM2(R47H) reduced phagocytosis by 50% and TREM2(Y38C) by 80% compared to wild-type TREM2.

To ensure co-expression of TREM2 and DAP12 in HEK293 cells, we inserted TREM2 and DAP12 cDNAs into a bicistronic vector, which contains 2A peptides to allow the co-expression of multiple genes from a single transcript using a single vector. We also established a stable cell line expressing TREM2 with the AD R47H mutation and DAP12. Anti-TREM2 and anti-DAP12 immunoblotting confirmed the co-expression of TREM2 and DAP12 in the HEK293 cells (Figure 1A). Laser confocal imaging following immunostaining with TREM2 and DAP12 antibodies reveals that HEK293 cells stably expressing DAP12 and TREM2 or the TREM R47H mutation display both cell surface and cytosolic expression of TREM2 as well as a colocalization between TREM2 and DAP12 for membrane associated TREM2 (Figure 1C). These data suggest that TREM2 and DAP12 undergo normal trafficking when stably expressed in HEK 293 cells and that the TREM2 R47H mutation does not impair the recruitment of DAP12 by TREM2. TREM2 R47H mutant has previously been shown to affect TREM2 maturation (Park et al., 2015, 2016; Kober et al., 2016; Sirkis et al., 2017). We confirmed this phenomenon, in our HEK293 cells expressing wild-type and mutant TREM2 by showing that the TREM2 R47H mutation reduces the maturation of TREM2 resulting in a greater amount of immature TREM2 and a lower level of mature TREM2 compared to cell expressing wild-type TREM2 (Figure 1A).

We further assessed the functionality of TREM2/DAP12 in HEK293 cell lines stably transfected with DAP12, wild-type TREM2 and TREM2 R47H by analyzing AKT activation by immunoblotting. We found that AKT phosphorylation at Ser473 was significantly increased in cells stably transfected with wild-type TREM2 and DAP12 but not in TREM2 R47H mutant cells suggesting a loss of function of the TREM2 R47H mutation as previously observed (Figures 1A,B). We next investigated whether the co-expression of TREM2-DAP12 was sufficient to transform HEK293 cells into phagocytic cells and whether the TREM2 R47H mutation was able to impact the phagocytosis activity of TREM2 as previously shown in myeloid cells (Kleinberger et al., 2014; Kober et al., 2016). Phagocytic activity was investigated by measuring the internalization of *E. coli* conjugated to a pH-sensitive pHrodo dye (pHrodo *E. coli*) that only yields a fluorescent signal in an acidic compartment. The phagocytosis of pHrodo *E. coli* bioparticles was evaluated in HEK293 cells expressing TREM2 only (TREM2), DAP12 (DAP12) only and in cells co-expressing TREM2 and DAP12 (TREM2 + DAP12). We show that the phagocytosis of *E. coli* bioparticles is only induced in HEK293 cells co-expressing TREM2 and DAP12 but is not observed in cells singly expressing either TREM2 or DAP12 showing that TREM2 dependent phagocytic activity requires DAP12 (Figure 1D). In addition, as a positive control, we used cytochalasin D (Cyto D), which inhibits phagocytosis in myeloid cells including microglia (N’Diaye et al., 2009) and demonstrated that Cyto D effectively abolishes the phagocytosis of pHrodo *E. coli* in TREM2 + DAP12 cells (Figure 1E). Expression of the AD-associated R47H variant and Nasu-Hakola disease (NHD)-associated TREM2 mutants Y38C also strongly reduced the phagocytic activity of TREM2 in HEK293 cells (Figure 1E) mimicking previous observations in microglial cells expressing TREM2 mutations (Kleinberger et al., 2014). Our data suggest that the co-expression of TREM2 and DAP12 in HEK293 cells is functional and can induce AKT activation which is one of the downstream TREM2 signaling molecule previously identified in immune cells (Peng et al., 2010). In addition, our HEK293 cell lines stably co-expressing DAP12 and wild-type TREM2 elicit phagocytic activity while the HEK293 cell lines expressing AD TREM2 R47H mutation or the NHD TREM2 Y38C demonstrate a reduced phagocytic activity reproducing previously described phagocytic dysfunction of TREM2 mutations observed in microglial cells (Kleinberger et al., 2014; Kober et al., 2016). Overall, these data suggest that our HEK293 cell lines co-expressing wild-type or mutant TREM2 with DAP12 constitute a suitable model to investigate TREM2 functions.

### TREM2 and DAP12 Coexpression Inhibits NFκB Activation in Response to PMA Treatment

**FIGURE 2:**
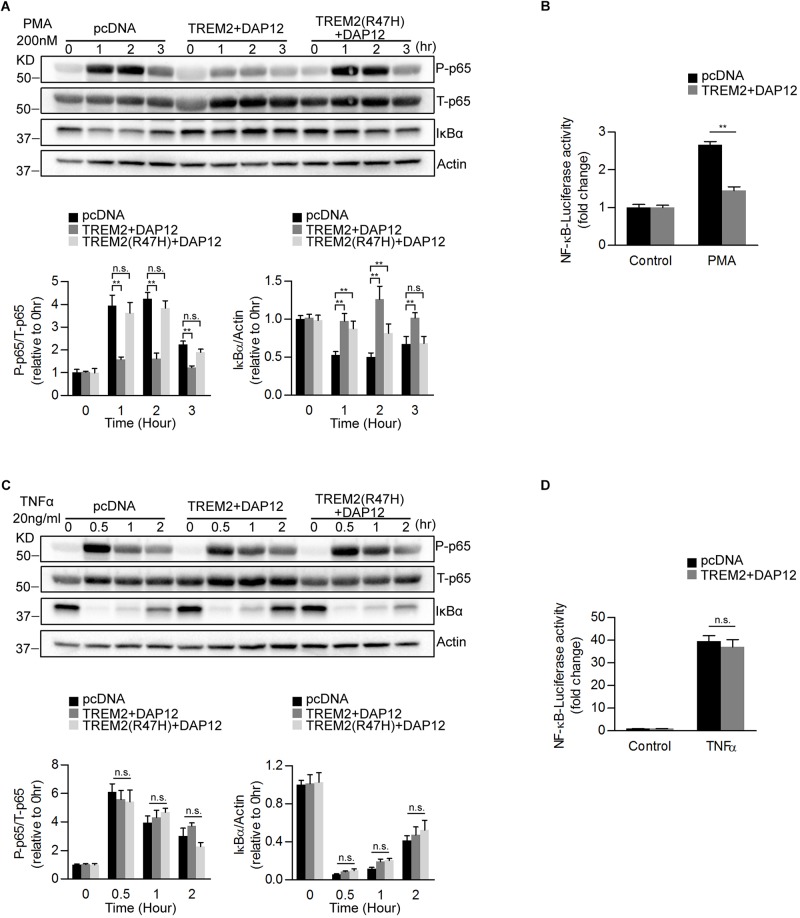
Overexpression of TREM2 and DAP12 in HEK 293 cells antagonizes PMA induced p65 NFκB phosphorylation and NFκB transactivity. **(A)** Western blot analysis of lysates from TREM2 + DAP12 and TREM2 (R47H) + DAP12 transfected HEK293 cells treated with PMA (200 nM) for different timepoints (0, 1, 2, and 3 h). Lower panel: the histogram represents the western-blot quantification of P-p65 NFκB (Ser536) normalized to total p65 NFκB (T-p65) levels and IκBα levels normalized to Actin levels. Data are represented as means ± SD and expressed relative to the 0-h timepoint; *n* = 4 for each experimental condition; statistical significance (^∗∗^*p* < 0.01; n.s., not significant) were evaluated by ANOVA followed by *post hoc* analyses with Bonferroni corrections. **(B)** The histogram represents the amount NFκB activation measured by quantifying PMA-induced intracellular luciferase activity in NFκB luciferase reporter HEK293 cell line transfected with pcDNA (empty vector) and TREM2 + DAP12. Data are represented as means ± SD from at least two independent cell culture experiments and expressed relative to NFκB luciferase activity measured in control cells transfected with the empty vector (pcDNA); *n* = 6 for each experimental condition; statistical significance (^∗∗^*p* < 0.01) was evaluated with ANOVA followed by *post hoc* analysis with Bonferroni corrections. **(C)** Western-blot analyses of lysates from TREM2 + DAP12 and TREM2 (R47H) + DAP12 transfected HEK293 cells treated with TNFα (20 ng/ml) for different timepoints (0, 0.5, 1, and 2 h). Lower panel: the histogram represents the western-blot quantification of P-p65 NFκB (Ser536)/total p65 NFκB (T-p65), and IκBα levels normalized to Actin. Data are represented as means ± SD and expressed relative to the 0-h timepoint; *n* = 4 for each experimental condition; statistical significance (n.s., not significant) was evaluated with ANOVA followed by *post hoc* analyses using Bonferroni correction. **(D)** The histogram represents the amount of NFκB activation measured by quantifying TNFα-induced intracellular luciferase activity in NFκB luciferase reporter HEK293 cells transfected with pcDNA (empty vector) and TREM2 + DAP12. Data are represented as means ± SD from at least two independent cell culture experiments and expressed relative to control HEK293 NFκB luciferase cells transfected with the empty vector; *n* = 6 for each experimental condition; statistical significance (n.s., not significant) was evaluated by ANOVA.

Previous studies have shown that TREM2 can modulate inflammatory signaling in immune cells including microglia (Hamerman et al., 2006; Turnbull et al., 2006). In particular, TREM2 has been shown to inhibit the NFκB pathway resulting in reduced pro-inflammatory cytokines production (Ren et al., 2018; Li et al., 2019). We therefore examined whether TREM2 and DAP12 co-expression was able to impact NFκB activation in our HEK293 cell lines. To induce NFκB, we treated HEK293 cells stably transfected with the pcDNA empty vector (pcDNA; control cells), TREM2 and DAP12 co-expression vector (TREM2 + DAP12) and TREM2 R47H mutant and DAP12 co-expression vector [TREM2(R47H) + DAP12] with Phorbol 12-myristate 13-acetate (PMA) and with TNFα. PMA treatment resulted in an increase in p65 NFκB (Ser536) phosphorylation and IκBα degradation in pcDNA cells but not in TREM2 + DAP12 cells, showing that TREM2 and DAP12 co-expression prevented NFκB activation in response to PMA treatment (Figure 2A). Interestingly, TREM2(R47H) + DAP12 cells showed an increased in p65 NFκB (Ser536) phosphorylation and IκBα degradation after PMA treatment, which was similar to that observed in control pcDNA cells. These results indicate that the AD-associated TREM2 R47H mutation results in a loss-of-function of TREM2 preventing TREM2 dependent inhibition of NFκB activation induced by PMA. We further examined the effect of TREM2 and DAP12 co-expression on PMA induced NFκB promoter luciferase activity in HEK293 NFκB -luciferase cells. We found that TREM2 and DAP12 overexpression significantly inhibited NFκB luciferase activation induced by PMA (Figure 2B) further confirming an inhibition of the NFκB pathway. By contrast, TNFα treatment increased p65 (Ser536) phosphorylation and IκBα degradation in all pcDNA, TREM2 + DAP12 and TREM2(R47H) + DAP12 cells (Figure 2C) to a similar extent showing that TREM2-DAP12 co-expression is unable to suppress NFκB activation induced by TNFα. This was further supported by the fact that TREM2 and DAP12 co-expression was also unable to block NFκB luciferase activation induced by TNFα in our NFκB luciferase reporter cells further suggesting that TREM2 does not affect the NFκB activation induced by TNFα (Figure 2D).

### Shedding of the TREM2 Ectodomain Does Not Prevent the Anti-inflammatory Function of TREM2

**FIGURE 3:**
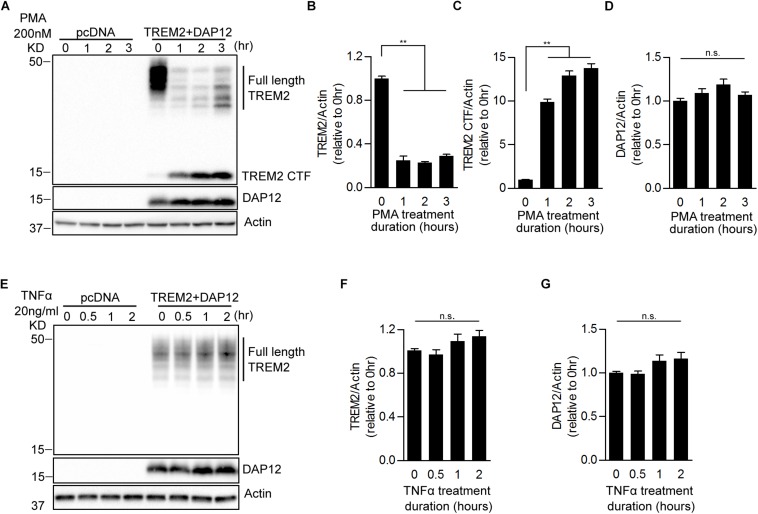
Effects of PMA and TNFα treatments on the shedding of TREM2 in HEK293 cells overexpressing TREM2 + DAP12. **(A)** A representative western-blot showing the effects of PMA on full length TREM2 and TREM2-CTF is shown. Control pcDNA (empty vector) and TREM2 + DAP12 transfected HEK293 cells were treated with PMA (200 nM) for different timepoints (0, 1, 2, and 3 h). The histograms represent the quantification of western-blot data and show the amount of full length TREM2 normalized to Actin **(B)**, TREM2-CTF levels normalized to Actin **(C)**, and DAP12 levels normalized to Actin **(D)**. Data are represented as means ± SD and expressed relative to the 0-h timepoint; *n* = 4 for each experimental condition; statistical significance (^∗∗^*p* < 0.01; n.s., not significant) was evaluated by ANOVA followed by *post hoc* analyses using Bonferroni corrections. **(E)** Western blot analysis of lysates from TREM2 + DAP12 transfected HEK293 cells treated with TNFα (20 ng/ml) for different timepoints (0, 0.5, 1, and 2 h). The histograms depict the quantification of full length TREM2 levels normalized to Actin **(F)** and DAP12 levels normalized to Actin **(G)**. Data are represented as means ± SD and expressed relative to 0-h timepoint; *n* = 4 for each experimental condition; statistical significance (n.s., not significant) was evaluated by ANOVA.

It has been shown recently that TREM2 is cleaved by ADAM10 and ADAM17 resulting in the production of soluble TREM2 (sTREM2) and a membrane-retained C-terminal fragment (TREM2-CTF) (Feuerbach et al., 2017; Schlepckow et al., 2017; Thornton et al., 2017). TREM2-CTF is then cleared by an intramembranous cleavage by the γ-secretase (Wunderlich et al., 2013). Since PMA is a strong activator of ADAM17, we investigated whether proteolytic processing of TREM2 was induced by PMA and determined the impact of TREM2 processing on phagocytic and anti-inflammatory functions of TREM2. We treated pcDNA and TREM2 + DAP12 cells with PMA and examined the levels of full length TREM2 (fTREM2) and of TREM2-CTF generated by ectodomain shedding at different time points. As expected, PMA treatment drastically lowered fTREM2 level and increased the generation of TREM2-CTF accordingly (Figures 3A–C). The level of DAP12 did not change after the PMA treatment (Figure 3D). We further examined the effect of TNFα treatment on TREM2 processing. We found that the levels of fTRM2 and DAP12 remained the same after TNFα treatment and that TREM2-CTF was not induced by the TNFα treatment showing that TNFα does not impact the processing of TREM2 (Figures 3E–G).

**FIGURE 4:**
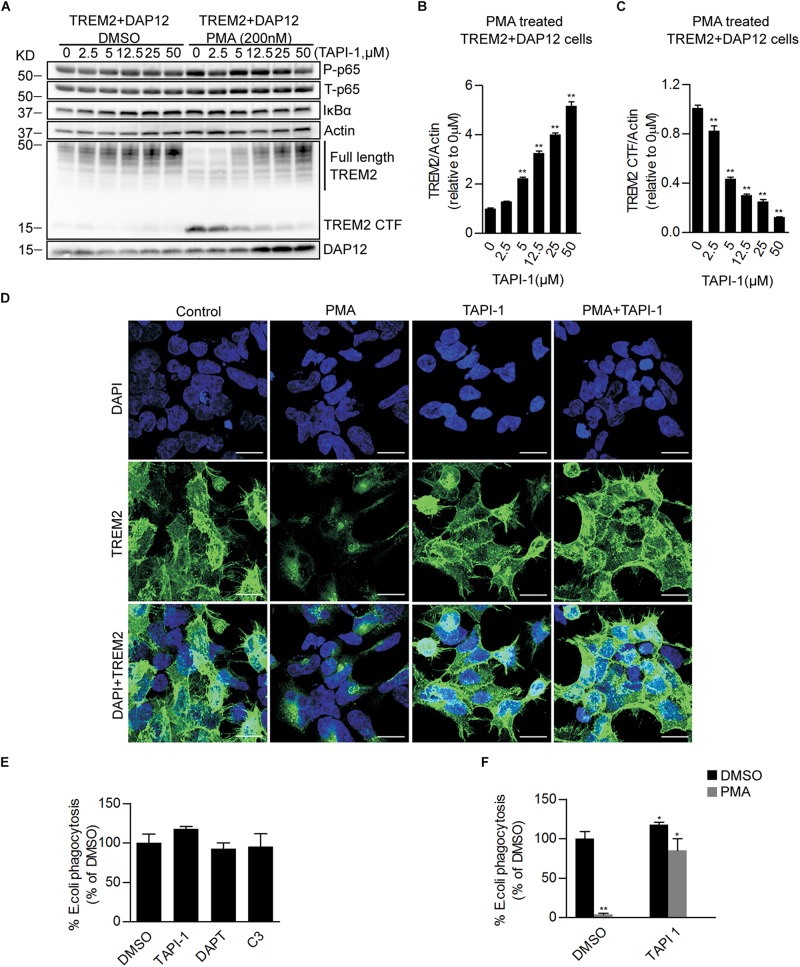
Pharmacological inhibition of ADAM proteases prevents PMA induced TREM2 shedding and increases phagocytosis in TREM2 + DAP12 HEK293 cells. **(A)** Western blot analysis of lysates from TREM2 + DAP12 HEK293 cells pretreated with different does of TAPI1 (0, 2.5, 5, 12.5, 25, and 50 μM) for 1 h, and then challenged with PMA (200 nM) for 1 h. The histograms represent the quantification of full length TREM2 levels normalized to Actin **(B)** and TREM2 CTF levels normalized to Actin used as a reference protein **(C)**. Data are represented as means ± SD and expressed relatively to the level of full length TREM2 quantified in TREM2 + DAP12 HEK293 cells prior to TAPI-1 treatment; *n* = 4 for each experimental condition; statistical significance (^∗∗^*p* < 0.01) was evaluated by ANOVA followed by *post hoc* analyses with Bonferroni corrections. **(D)** Representative laser confocal images obtained following immunostaining with an N-terminal TREM2 antibody (green) and counterstaining with DAPI (blue) in HEK293 cells transfected with TREM2 + DAP12. Cells were pretreated with or without TAPI 1 (25 μM) for 1 h, and then challenged with PMA (200 nM) for 1 h prior to immunostaining. The white scale bar represents 20 μm. **(E)** Phagocytosis of pHrodo *E. coli* bioparticles conjugate in TREM2 + DAP12 transfected HEK293 cells treated with TAPI 1 (25 μM), DAPT (10 μM), or C3 (10 μM) for 1 h. Data are represented as means ± SD from at least two independent cell culture experiments and expressed as a percentage of the values obtained in DMSO (vehicle used to dissolve TAPI-1, DAPT, and C3) treatment conditions (control); *n* = 6 for each experimental condition; statistical significance (^∗^*p* < 0.05) was evaluated by ANOVA followed by *post hoc* analyses with Bonferroni corrections. **(F)** Phagocytosis of pHrodo *E. coli* bioparticles conjugate in TREM2 + DAP12 transfected HEK293 cells pretreated with TAPI-1 (25 μM) for 1 h and then challenged with PMA (200 nM) for 1 h. Data are represented as means ± SD from at least two independent cell culture experiments and expressed as a percentage of the values obtained in TREM2 + DAP12 HEK293 cells following DMSO treatment; *n* = 6 for each experimental condition; statistical significance (^∗^*p* < 0.05; ^∗∗^*p* < 0.01) was evaluated by ANOVA followed by *post hoc* analyses with Bonferroni correction.

We further examined the effect of a blockade of the proteolytic processing of TREM2 on the anti-inflammatory functions of TREM2 by using the broad-spectrum inhibitor of the ADAM family of proteases, tumor necrosis factor-α protease inhibitor (TAPI-1) in TREM2 + DAP12 cells. TAPI-1 treatment was able to effectively increase the steady state level of TREM2 in TREM2 + DAP12 cells, indicating that TAPI-1 is blocking the shedding of fTREM2 (Figure 4A). TAPI-1 also blocked the effects of the PMA treatment on fTREM2 degradation and TREM2-CTF generation in a dose dependent manner but did not affect the inhibition of p65 NFκB(Ser536) phosphorylation and IκBα degradation in TREM2 + DAP12 cells (Figures 4A–C). Moreover, we confirmed by laser confocal imaging following TREM2 immunostaining with an antibody detecting the N-terminal domain of TREM2 that TAPI-1 protected TREM2 from the shedding induced by PMA treatment in TREM2 + DAP12 cells (Figure 4D). These results suggest that blocking the ectodomain shedding of TREM2 with TAPI-1 does not affect the anti-inflammatory functions of TREM2. The fact that the NFκB activation induced by PMA in TREM2-DAP12 co-expressing cells is blocked even after complete shedding of TREM2 suggest that the anti-inflammatory functions of TREM2 may be exerted by TREM2-CTF under these circumstances and therefore do not require the N-terminal domain of TREM2.

We also investigated the effects of an inhibition of the proteolytic processing of TREM2 on TREM2 phagocytic functions. We found that TAPI-1 was significantly increasing the phagocytosis of pHrodo *E. coli*, whereas PMA was completely antagonizing TREM2 dependent phagocytic activity (Figures 4E,F). Interestingly, the amount of phagocytosis stimulation induced by TAPI-1 in our TREM2 + DAP12 HEK293 cells is similar to the increased in phagocytosis induced by a broad-spectrum ADAM protease inhibitor (GM6001) in a microglial cell line (Kleinberger et al., 2014). We also tested in the phagocytosis assay the impact of DAPT, an inhibitor of the γ-secretase which has been shown previously to prevent the clearance of the TREM2-CTF (Feuerbach et al., 2017) and of C3, an inhibitor of the β-secretase (Kleinberger et al., 2014). We found that DAPT did not affect the phagocytosis of pHrodo *E. coli* in TREM2 + DAP12 cells (Figure 4E) suggesting that TREM2 dependent phagocytosis is not influenced by the γ-secretase processing of TREM2-CTF. The increased phagocytosis induced by TAPI-1 is paralleled by an increased in fTREM2 levels observed after TAPI-1 treatment. We found that TAPI-1 was able to completely reverse the inhibition of TREM2 dependent phagocytosis induced by PMA as well as the shedding of TREM2 in TREM2 + DAP12 cells indicating that the phagocytic functions of TREM2 are correlated with the levels of fTREM2 and require fTREM2.

### APOE Induces SYK Activation in a TREM2 Dependent Manner

**FIGURE 5:**
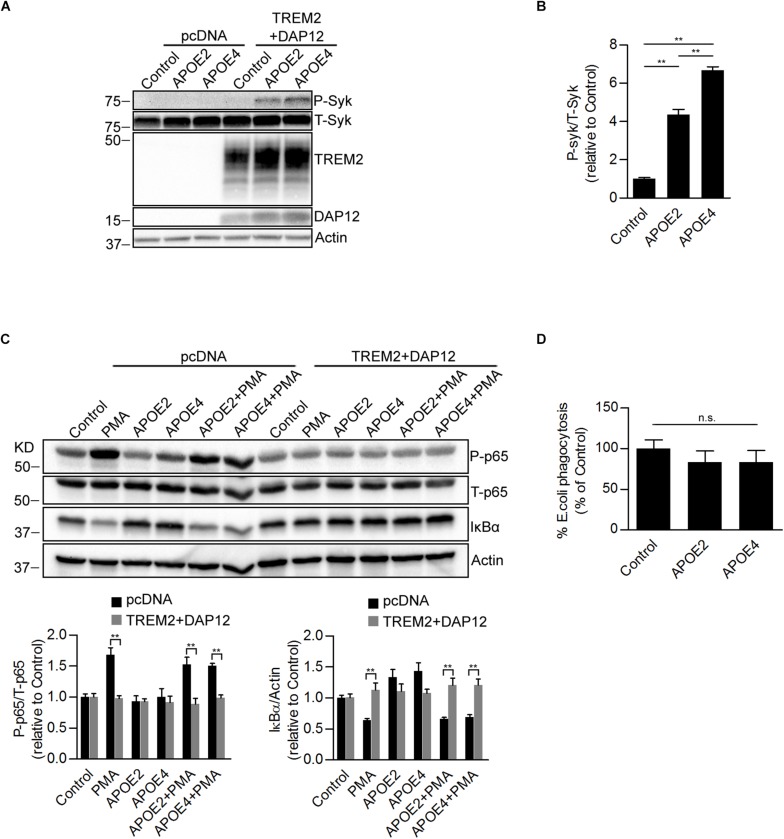
Effects of ApoE isoforms on SYK kinase phosphorylation, *NF*-κ*B* activation, and phagocytosis in TREM2 + DAP12 transfected HEK293 cells. **(A)** Western blot analysis of lysates from pcDNA and TREM2 + DAP12 transfected HEK293 cells following treatment with APOE2 (10 μg/ml) and APOE4 (10 μg/ml) for 12 h. **(B)** The histogram represents the quantification of P-Syk (Tyr525/526)/total Syk(T-Syk) ratios in TREM2 + DAP12 HEK293 cells with and without treatment with ApoE isoforms. Data are represented as means ± SD and expressed relative to TREM2 + DAP12 cells untreated with ApoE isoforms; *n* = 4 for each treatment condition; statistical significance (^∗∗^*p* < 0.01) was evaluated by ANOVA followed by *post hoc* analyses with Bonferroni correction. **(C)** Western blot analysis of lysates from pcDNA (empty vector) or TREM2 + DAP12 transfected HEK293 cells pretreated with APOE2 (10 μg/ml) and APOE4 (10 μg/ml) for 12 h, and then challenged with PMA (200 nM) for 1 h. Lower panel: the histograms represent the quantification of P-p65 (Ser536)/total P65 (T-p65) ratios and IκBα levels normalized to Actin. Data are represented as means ± SD and expressed relative to untreated pcDNA (empty vector) control cells; *n* = 4 for each treatment condition; statistical significance (^∗∗^*p* < 0.01) was evaluated by ANOVA followed *post hoc* analysis with Bonferroni correction. **(D)** Phagocytosis of pHrodo *E. coli* bioparticles conjugate in TREM2 + DAP12 transfected HEK293 cells pretreated with APOE2 (10 μg/ml) and APOE4 (10 μg/ml) for 12 h. Data are represented as means ± SD from at least two independent cell culture experiments and expressed as a percentage of the phagocytic activity measured in TREM2 + DAP12 HEK293 cells without APOE treatment; *n* = 6 for each experimental condition; ANOVA reveals no significant main effect of APOE4 and APOE2 on phagocytosis (n.s., not significant).

It has been reported that TREM2 binds to apolipoproteins including APOE, which is the strongest genetic risk factor for late-onset Alzheimer disease. We next investigated whether APOE was able to affect TREM2 signaling. It has been shown that upon TREM-2 binding with a TREM2 activating antibody that tyrosine residues in the ITAM domain of DAP12 are phosphorylated allowing the recruitment and activation of the SYK in myeloid cells (Yeh et al., 2017). We therefore examined whether APOE2 and APOE4 were able to induce an activation of SYK in HEK293 cells co-expressing TREM2 and DAP12. We show that both APOE2 and APOE4 effectively activates SYK in HEK293 cells stably overexpressing TREM2 and DAP12 (Figures 5A,B). This effect of APOE was mediated by TREM2 since it was not observed in cells that did not express TREM2 (pcDNA empty vector control cells). These data therefore confirm in our model that APOE is an agonist of TREM2. Interestingly, our data suggest that APOE4 is able to induce SYK activation more potently than APOE2 (Figures 5A,B), supporting a differential effect of ApoE isoforms on TREM2 signaling. We further examined whether APOE was affecting NFκB activation and phagocytosis in TREM2 + DAP12 cells. We found that APOE2 and APOE4 had no effect on PMA induced NFκB activation in pcDNA and did not block the anti-inflammatory activity of TREM2 in TREM2 + DAP12 cells (Figure 5C). In the phagocytosis assay, TREM2 + DAP12 cells showed a decreasing trend in phagocytic activity after APOE2 and APOE4 treatment (Figure 5D). Overall these results show that APOE does not affect the anti-inflammatory and has only a marginal impact on the phagocytic activity of TREM2 in TREM2 + DAP12 cells.

### SYK Kinase Is Required for TREM2 Dependent Phagocytosis but Not for the Suppression of NFκB Activation

**FIGURE 6:**
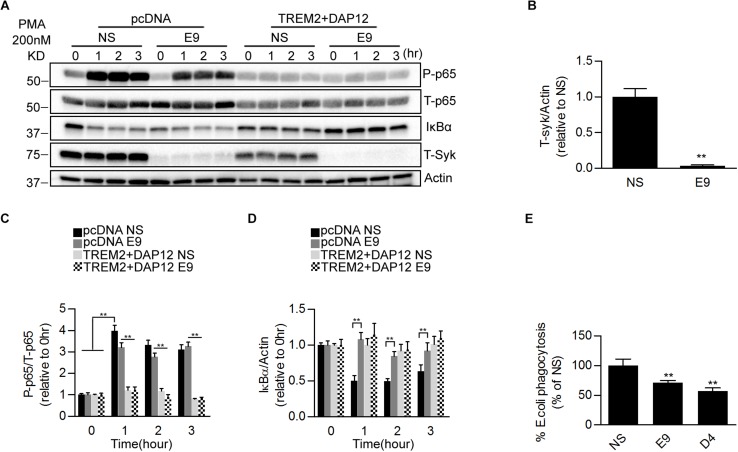
Effects of genetic suppression of SYK kinase expression on *NF*κ*B* activation and phagocytosis in TREM2 + DAP12 transfected HEK293 cells. **(A)** Western blot analysis of lysates from pcDNA or TREM2 + DAP12 transfected HEK293 cells, with or without silencing of the Syk gene [lentiviral vector expressing Syk specific shRNA(E9) or nonsense control shRNAs(NS)], following treatment with PMA (200 nM) for the indicated timepoints (0, 1, 2, and 3 h). **(B)** The histogram represents the quantification of Syk levels normalized to Actin in control TREM2 + DAP12 HEK293 cells transfected with the nonsense shRNA (NS) and in TREM2 + DAP12 cells in which the Syk gene was silenced (shRNA E9). Data are represented as means ± SD and expressed relative to RNAi the amount of SYK quantified in shRNA nonsense (NS) transfected cells; *n* = 4 for each experimental condition; statistical significance (^∗∗^*p* < 0.01) was evaluated with two-tailed Student’s *t*-test. **(C,D)** The histograms represent the ratios of P-p65 NFκB (Ser536)/total p65 NFκB (T-p65) and IκBα levels normalized to Actin. Data are represented as means ± SD and expressed relative to pcDNA (empty vector) shRNA nonsense (NS) transfected cells; *n* = 4 for each treatment condition; statistical significance (^∗∗^*p* < 0.01; n.s., not significant) was evaluated by ANOVA followed by *post hoc* analyses with Bonferroni correction. **(E)** Phagocytosis of pHrodo *E. coli* bioparticles conjugate in TREM2 + DAP12 transfected HEK293 cells with or without silencing of the Syk gene [lentiviral vector expressing Syk specific shRNA (E9 and D4) or nonsense control shRNAs(NS)]. Data are represented as means ± SD from at least two independent cell culture experiments and expressed relative to shRNA nonsense (NS) transfected cells TREM2 + DAP12 HEK293 cells; *n* = 6 for each experimental condition; statistical significance (^∗∗^*p* < 0.01) was evaluated by ANOVA followed by *post hoc* analyses with Bonferroni correction.

We further examined the possible contribution of SYK in regulating the phagocytic and the inhibition of NFκB activation by TREM2. We used short hairpin RNAs (shRNAs) to stably knockdown the SYK gene expression in pcDNA and TREM2 + DAP12 cells (Figures 6A,B). We then measured the effect of SYK knockdown on PMA-induced NFκB activation and phagocytosis of pHrodo *E. coli*. We found that the suppression of SYK expression was slightly reducing PMA stimulation of NFκB p65 phosphorylation in pcDNA cells but had no effect on the inhibition of p65 NFκB phosphorylation in TREM2 + DAP12 cells with or without PMA treatment (Figures 6C,D). Interestingly, the phagocytic activity of TREM2 was partially prevented by the suppression of SYK expression (Figure 6E). Collectively, these data suggest that the inhibition of NFκB activation by TREM2 is SYK independent whereas its phagocytic functions are partially mediated via a SYK dependent pathway.

### PI3K/AKT Signaling Differently Affects the Anti-inflammatory and Phagocytic Functions of TREM2

**FIGURE 7:**
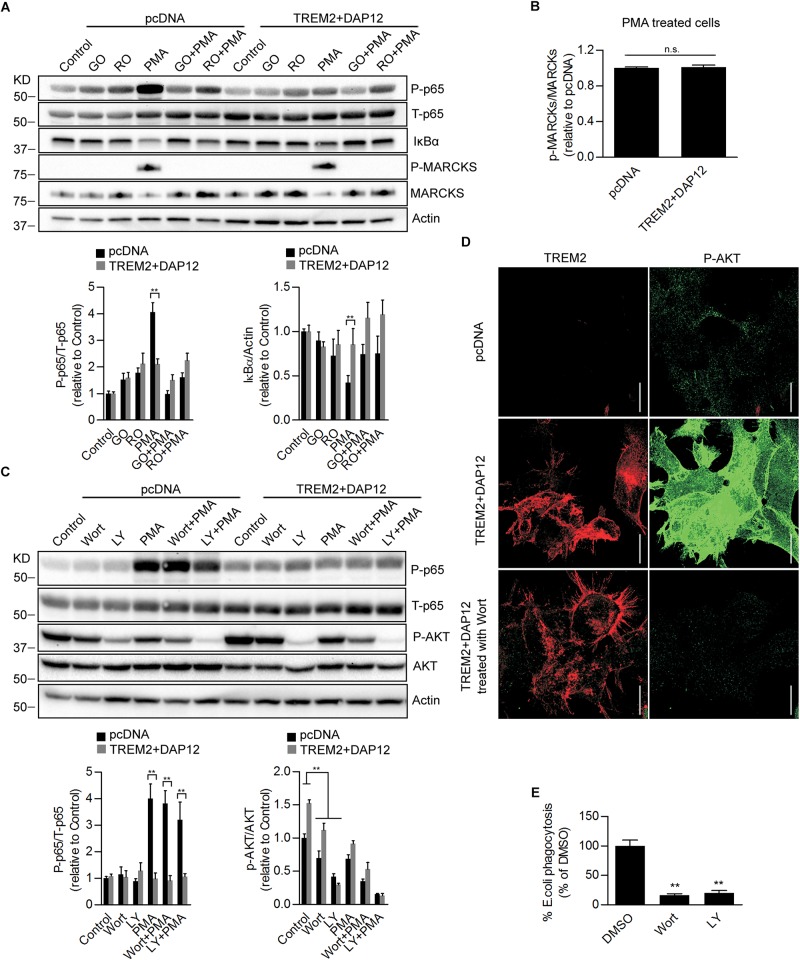
Effects of Pharmacological inhibition of PI3K/AKT signaling on *NF*κ*B* activation and phagocytosis in TREM2 + DAP12 HEK293 cells. **(A)** Western-blot analysis of lysates from TREM2 + DAP12 transfected HEK293 cells pretreated with different PKC inhibitors Go 6983 (Go; 5 μM), Ro 32-0432 (Ro; 10 μM) for 1 h, and then challenged with PMA (200 nM) for 1 h. Lower panel: the histograms represent the ratios of P-p65 NFκB (Ser536)/total p65 NFκB and IκBα levels normalized to Actin in control pcDNA (empty vector) cells and in TREM2 + DAP12 HEK93 cells. Data are represented as means ± SD and expressed relative to control pcDNA cells; *n* = 4 for each experimental condition; statistical significance (^∗∗^*p* < 0.01) was evaluated by ANOVA followed by *post hoc* analyses with Bonferroni correction. **(B)** The histogram represents the ratios of p-MARCKs/total MARCKs in PMA treated pcDNA (control cells) and TREM2 + DAP12 transfected HEK293 cells. Data are represented as means ± SD and expressed relative to pcDNA transfected cells; *n* = 4 for each experimental condition; no statistical significance (n.s.; *p* > 0.05) was found using a two-tailed Student’s *t*-test. **(C)** Western blot analysis of lysates from pcDNA (empty vector) and TREM2 + DAP12 transfected HEK293 cells pretreated with different PI3K inhibitors: wortmannin (wort; 200 nM) and LY 294002 (LY; 20 μM) for 1 h, and then challenged with PMA (200 nM) for 1 h. Lower panel: the histograms represent the ratios of P-p65 NFκB (Ser536)/total p65 NFκB (T-p65) and p-AKT(Ser473)/Total AKT levels normalized to Actin quantified in control pcDNA (empty vector) and TREM2 + DAP12 HEK293 cells. Data are represented as means ± SD and expressed relative to control pcDNA (empty vector) transfected HEK293 cells; *n* = 4 for each experimental condition; statistical significance (^∗∗^*p* < 0.01) was evaluated by ANOVA followed by *post hoc* analyses with Bonferroni corrections. **(D)** Representative laser confocal images obtained following immunostaining of HEK293 cells transfected with pcDNA (empty vector) and TREM2 + DAP12 HEK293 cells using an N-terminal TREM2 antibody (red), and a p-AKT (Ser473) antibody (green) following treatment of the cells with the PI3K inhibitor wortmannin (wort; 200 nM) for 1 h. The white scale bar represents 20 μm. **(E)** Phagocytosis of pHrodo *E. coli* bioparticles conjugate in TREM2 + DAP12 transfected HEK293 cells treated with the PI3K inhibitors wortmannin (wort; 200 nM) and LY 294002 (LY; 20 μM) for 1 h. Data are represented as means ± SD from at least two independent cell culture experiments and expressed relative to DMSO (vehicle) conditions in TREM2 + DAP12 HEK293 cells; *n* = 6 for each treatment condition; statistical significance (^∗∗^*p* < 0.01) was evaluated by one-way ANOVA followed by *post hoc* analyses with Bonferroni corrections.

PMA is a diacylglycerol (DAG) analog known to activate protein kinase C (PKC) resulting in the activation of the NFκB pathway and inflammation (Holden et al., 2008). Our data therefore suggest that inhibition of NFκB activation induced by TREM2 in response to PMA is mediated by a suppression of the PKC pathway. To investigate whether PKC activation induced by PMA was affected in TREM2 + DAP12 cells, we monitored the phosphorylation of Myristoylated Alanine Rich Protein Kinase C Substrate (MARCKs), the most prominent cellular substrate for PKC. In addition, we also inhibited PKC using two different PKC inhibitors, Go 6983 or Ro 32-0432. We found as expected that PKC inhibitors can totally block PMA-induced NFκB activation and phosphorylation of MARCKs in pcDNA cells (Figure 7A) showing that the stimulation of NFκB induced by PMA is effectively driven by an activation of PKC enzymes. There was however no difference in the phosphorylation of MARCKs between pcDNA and TREM2 + DAP12 cells after PMA treatment, indicating that PKC activation was similar in pcDNA and TREM2 + DAP12 cells (Figure 7B) suggesting that TREM2 is acting downstream of PKC to block NFκB activation.

As shown earlier, AKT activation was observed in TREM2 + DAP12 cells, which is consistent with previous reports demonstrating an activation of the PI3K/AKT pathway by TREM2 in myeloid cells (Peng et al., 2010). To examine the role of PI3K/AKT signaling on TREM2 functions, we used two different PI3K inhibitors, wortmannin and LY294002. As shown by immunoblotting and immunostaining, AKT phosphorylation was effectively blocked by the two inhibitors of PI3K in TREM2 + DAP12 cells confirming that TREM2 activates PI3K (Figures 7C,D). Interestingly, inhibiting PI3K/AKT signaling had no effect on the inhibition of NFκB activation induced by TREM2 since PMA-induced NFκB activation was still suppressed in TREM2-DAP12 cells when PI3K was inhibited (Figure 7C). By contrast, inhibition of PI3K/AKT signaling largely decreased the phagocytosis of pHrodo *E. coli* in TREM2 + DAP12 cells, suggesting that phagocytic functions of TREM2 require PI3K/AKT signaling (Figure 7E).

## Discussion

TREM2 signals through the intracellular adaptor DAP12 (Lanier et al., 1998; Bouchon et al., 2001; Daws et al., 2001) also known as TYRO protein- TYROBP. TREM2 ligation promotes the phosphorylation of its ITAMS by Src kinases creating a docking site for the SH2 domains of several proteins including SYK (Takahashi et al., 2005; Mocsai et al., 2010; Yu et al., 2013). Upon ligation of TREM2, downstream signaling molecules including phosphatidyl inositol 3-kinase (PI3K) and phospholipase Cγ (PLCγ) have been shown to be activated (Takahashi et al., 2005; Otero et al., 2009; Peng et al., 2010; Wang et al., 2015; Colonna and Wang, 2016; Zhao et al., 2018). It remains unclear how TREM2 exerts an anti-inflammatory activity as TREM2 ligands are known to promote SYK signaling which has been shown to mediate inflammation notably in microglia and myeloid cells (McDonald et al., 1997; Combs et al., 2001; Sondag et al., 2009; Zeng et al., 2014; Xie et al., 2017). TREM2 ligation with Aβ oligomers has been shown to promote SYK activation and NFAT signaling (Lessard et al., 2018). The activation of NFAT signaling could be mediated by SYK which is known to activate the PLCγ-calcineurin-NFAT pathway (Mocsai et al., 2010). We therefore investigated here whether SYK, PLCγ, and PI3K were required to mediate the inhibition of NFκB activation and phagocytic functions of TREM2. In addition, we tested the impact of TREM2 ligation with ApoE2 and ApoE4 isoforms on SYK and NFκB activation.

Our data show that TREM2 inhibits NFκB activation only when DAP12 is co-expressed with TREM2. Similarly, we show that TREM2 dependent phagocytic activity also requires DAP12. Interestingly, HEK293 cells co-expressing the AD variant TREM2 R47H with DAP12 are unable to suppress NFκB activation induced by PMA and to phagocyte *E. coli* bioparticles suggesting that the TREM2 R47H variant confers loss-of-function-like phenotypes. Our data further suggest that the inhibition of NFκB activation by TREM2/DAP12 originates from a decreased degradation of IκBα (which inhibits NFκB). Following PMA stimulation, IκBα degradation and NFκB activation induced by PMA are not inhibited by the TREM2 AD variant R47H suggesting a loss of TREM2 function. We show that PMA stimulates PKC activity to a similar extent in TREM2/DAP12 overexpressing cells and control HEK293 cells as the phosphorylation of myristoylated Alanine-rich C Kinase substrate (MARCKS), substrate of PKC, was similar in these cells. As expected, PKC inhibition was able to prevent NFκB activation induced by PMA in control HEK293 cells and did not suppress the inhibition of NFκB activation in TREM2/DAP12 overexpressing cells. These data therefore suggest that TREM2/DAP12 antagonizes signaling elements downstream of PKC that are required to mediate NFκB activation. For instance, PKC has been shown to direct the assembly of the CARMA1-BCL10-MALT1 (CBM complex) which is essential to mediate NFκB activation and JNK signaling pathways (Matsumoto et al., 2005; Hara et al., 2008; So et al., 2011). It is interesting to note that both JNK and NFκB signaling have been shown to be affected by TREM2/DAP12 in microglia (Zhong et al., 2017b) which could result from a diminished CBM complex formation. More work will be required to determine whether TREM2/DAP12 can effectively impact the formation of the CBM complex. Interestingly, genetic variations that enhance PKCα activity have been identified in patients with AD (Alfonso et al., 2016) whereas inhibition of PKC has been shown to reduce amyloid-β levels and neuroinflammation in an AD mouse model (Du et al., 2018) suggesting that PKC activation has a deleterious impact in AD. Our data showing that TREM2 can negate PKC induced NFκB activation could therefore support a positive role of TREM2 for alleviating neuroinflammation triggered by PKC stimulation in AD.

The inhibition of NFκB activation by TREM2/DAP12 in response to PMA was observed without the need to add an exogenous ligand for TREM2 which could suggest that TREM2 ligation is not required for its anti-inflammatory activity or that HEK293 cells produce an endogenous TREM2 ligand. We found that PMA induces a rapid cleavage of full length TREM2 resulting in a decreased cell surface expression of the TREM2 N-terminal domain and an increased production of the TREM2 C-terminal fragment (TREM2-CTF) while the inhibition of NFκB activation was still observed. These data further suggest that the N-terminal portion of the TREM2 receptor is not required to mediate the anti-inflammatory activity of TREM2 and therefore that TREM2 ligation is not necessary for that effect. TREM2 N-terminal domain undergoes shedding by proteases in the ADAM (a disintegrin and metalloproteinase) family including ADAM10 and ADAM17 (Kleinberger et al., 2014; Feuerbach et al., 2017; Schlepckow et al., 2017; Thornton et al., 2017) resulting in the production of soluble TREM2 (sTREM2) and TREM2-CTF. sTREM2 has been postulated to act as a decoy receptor that could bind TREM2 ligands and antagonize TREM2 signaling (Piccio et al., 2008; Zhong et al., 2017a). We found that in presence of the ADAM10/ADAM17 inhibitor TAPI-1 (which blocked the shedding of TREM2 following PMA treatment) that there was no modulation of the TREM2/DAP12 inhibition of NFκB activation suggesting that both the full length TREM2 receptor and the TREM2-CTF are capable of inhibiting NFκB. These data further imply that TREM2 suppression of NFκB activation is not dependent on the presence of sTREM2 as TAPI-1 treatment which inhibits the production of sTREM2 did not affect the inhibition of NFκB by TREM2. It has been shown that TREM2-CTF inhibits LPS induced inflammation in microglial cells (Zhong et al., 2015) showing also that the full-length receptor and TREM2 ligation are not required for the anti-inflammatory activity of TREM2 in myeloid cells which is consistent with our observations.

We found that TREM2/DAP12 overexpression led to a stimulation of AKT phosphorylation which was blocked by the PI3K inhibitors wortmannin and LY294002 showing that the PI3K/AKT pathway is triggered by TREM2/DAP12. We found however that there was no activation of PI3K/AKT in cells co-expressing the TREM2 AD mutation R47H with DAP12. Additionally, we observed that PI3K inhibition is unable to suppress the inhibition of NFκB activation following PMA stimulation in TREM2/DAP12 cells showing that the PI3K/AKT activation is probably not responsible for the inhibition of NFκB activation in TREM2/DAP12 cells.

**FIGURE 8:**
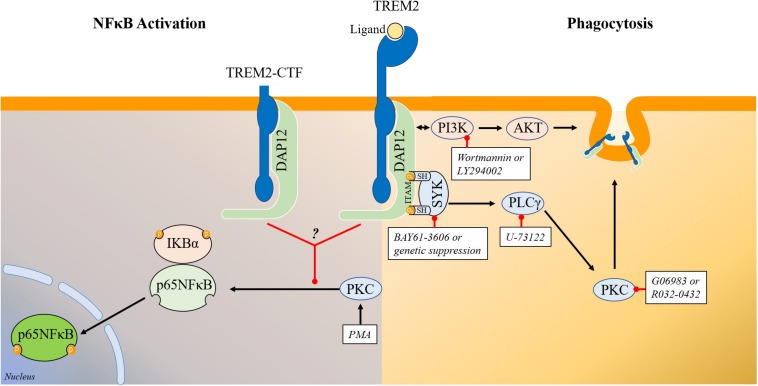
Schematic representation of the signaling pathways regulating TREM2 phagocytosis and TREM2 modulation of NFκB phosphorylation/activation. **(Right)** Schematic representation of the signaling pathways involved in the regulation of phagocytosis by TREM2. Overall our data show that TREM2 leads to an activation of SYK and of the PI3K/AKT pathways. Genetic suppression or inhibition of SYK activity with BAY61-3606 does not prevent the induction of the PI3K/AKT pathway in HEK293 TREM2/DAP12 cells suggesting that SYK activation and PI3K activation are triggered independently by TREM2. The PI3K inhibitors wortmannin and LY294002 prevent AKT phosphorylation in TREM2/DAP12 cells showing that the TREM2 stimulation of AKT phosphorylation is mediated by PI3K while suppression of SYK expression as well as SYK inhibition with BAY61-3606 do not affect AKT phosphorylation. Interestingly, both SYK inhibition with BAY61-3606 or suppression of SYK expression, PI3K inhibition with wortmannin and LY294002, PLCγ inhibition with U-73122 and PKC inhibition with Go6983 and Ro32-0432 are all antagonizing TREM2 phagocytosis in HEK293 TREM2-DAP12 cells highlighting the involvement of these signaling elements in TREM2 mediated phagocytosis. **(Left)** Schematic representation of signaling elements involved in the regulation of p65NFκB phosphorylation by TREM2. TREM2-DAP12 prevents the degradation of IKBα induced by PMA, suppresses p65 NFκB phosphorylation induced by PMA and also prevents NFκB transactivation induced by PMA showing globally that TREM2-DAP12 antagonizes NFκB activation induced by PMA. Overall, the suppression of p65NFκB phosphorylation by TREM2 (either full length or TREM2-CTF) in response to PMA is not affected by SYK inhibition, PI3K inhibition or PLCγ inhibition. In addition, MARCKS phosphorylation (direct substrate of PKCs) induced by PMA is not suppressed by TREM2 showing that TREM2 does not prevent PKC activation induced by PMA. These data suggest that TREM2 is acting downstream of PKC to suppress p65NFκB phosphorylation induced by PMA.

TREM2 ligation with a stimulating antibody or with Aβ oligomers has been shown to trigger SYK activation (Zhao et al., 2018; Zhong et al., 2018). However, SYK activation has been shown to promote inflammation and NFκB activation (McDonald et al., 1997; Combs et al., 2001; Sondag et al., 2009) while SYK inhibition is anti-inflammatory (Zeng et al., 2014; Xie et al., 2017; Sahan-Firat et al., 2019). These data could therefore suggest that upon ligation, TREM2 may have a proinflammatory activity and therefore that TREM2 could have both anti-inflammatory and pro-inflammatory functions depending on the presence and nature of TREM2 ligands. For example, intrathecal injection of an agonistic TREM2 antibody has been shown to result in microglial activation and increased proinflammatory cytokines production (Kobayashi et al., 2016). Similarly, myelin lipids following demyelination have been shown to trigger TREM2 signaling resulting in microglial activation and upregulation of proinflammatory genes (Cantoni et al., 2015; Poliani et al., 2015) which could support that hypothesis. It remains also possible that different TREM2 ligands may have different activities toward TREM2 signaling as previously postulated (Turnbull et al., 2006; Kober and Brett, 2017). To further analyze the possible involvement of SYK in the regulation of NFκB activation in TREM2/DAP12 cells, we generated TREM2/DAP12 overexpressing HEK293 cells that are also knockdown for SYK. We found that in absence of SYK expression, TREM2/DAP12 still dampen PMA induction of NFκB activation showing that SYK is not required to mediate TREM2 suppression of NFκB activation. In cells that do not express TREM2/DAP12, we observed that downregulation of SYK expression was partially suppressing PMA induction of p65NFκB phosphorylation further confirming a pro-inflammatory function of SYK. In addition, we found that PI3K/AKT stimulation induced by TREM2/DAP12 overexpression was not suppressed in SYK knockdown cells showing that SYK does not mediate the PI3K/AKT stimulation observed in TREM2/DAP12 overexpressing cells which could suggest that DAP12 could also directly recruit PI3K independently of SYK as previously thought (Peng et al., 2010). We tested the possible contribution of PLCγ in mediating the anti-inflammatory activity of TREM2 as previous data have suggested that TREM2 ligation can activate PLCγ (Takahashi et al., 2005; Otero et al., 2009; Peng et al., 2010; Wang et al., 2015; Colonna and Wang, 2016; Zhao et al., 2018). We found that PLCγ inhibition has no impact on the suppression of NFκB activation observed in TREM2/DAP12 overexpressing cells following challenge with PMA. In addition, PLCγ inhibition did not impact NFκB activation induced by PMA in control HEK293 cells further suggesting that the inhibition of NFκB activation observed in TREM2/DAP12 co-expressing cells following PMA challenge is PLCγ independent. We evaluated the possible effect of APOE on NFκB in TREM2/DAP12 as we have shown that APOE stimulation of TREM2 results in the activation of SYK. We observed that APOE did not trigger NFκB activation or affect basal p65NFκB phosphorylation in TREM2/DAP12 cells. Overall, these data show that the inhibition of NFκB activation by TREM2/DAP12 is SYK, PLCγ, and PI3K/AKT independent and does not require the stimulation of TREM2 by a ligand. Figure 8 provides a schematic representation of the signaling pathways regulating TREM2 phagocytosis and TREM2 suppression of NFκB activation.

Protein binding assays suggest that APOE binds to TREM2 (Atagi et al., 2015; Bailey et al., 2015; Yeh et al., 2016) raising the possibility that APOE-TREM2 interactions may trigger TREM2 signaling. We analyzed this possibility in HEK293 cells expressing TREM2/DAP12 and TREM2 R47H/DAP12 constructs. We show that treatment of TREM2/DAP12 overexpressing cells with APOE induces SYK activation. Interestingly, APOE4 appears to stimulate SYK activation with greater potency than APOE2. This effect of APOE is TREM2 mediated as no stimulation of SYK phosphorylation by APOE was observed in control HEK293 cells that do not express TREM2. In cells expressing the TREM2 AD variant R47H, APOE was still able to stimulate SYK phosphorylation although to a lesser extent than in wild-type TREM2 expressing cells suggesting a subtle loss of function of TREM2 R47H for that particular outcome.

We found that TREM2 phagocytosis was partially prevented in SYK knockdown cells and was antagonized by PI3K inhibition. Although treatment of TREM2/DAP12 cells with APOE stimulates SYK activation, APOE isoforms do not appear to significantly impact the phagocytosis of *E. coli* bioparticles. In addition, we found that PLCγ inhibition potently suppresses TREM2/DAP12 phagocytic activity. These data therefore demonstrate that TREM2/DAP12 phagocytic and anti-inflammatory activities are mediated by distinct signaling pathways. Interestingly, stimulation of TREM2 processing by PMA completely antagonizes TREM2 dependent phagocytosis. A phenomenon which was reverted using the ADAM10/ADAM17 inhibitor TAPI-1 showing that full length TREM2 receptor is required for its phagocytic activity.

HEK293 are not immune cells and therefore may not react like immune cells to inflammatory challenges. This is a limitation of the ectopic HEK293 cell model to study TREM2 biology and the findings observed in this cellular model will require validation in immune cells such as microglia cells. In summary, our data show that TREM2 suppression of NFκB activation induced by PMA is not mediated by a stimulation of the PI3K/AKT pathway which occurs following TREM2/DAP12 overexpression. In addition, our data suggest that TREM2 ligation, PLCγ, SYK or the N-terminal domain of TREM2 are not necessary to mediate the suppression of NFκB activation mediated by TREM2 whereas TREM2 dependent phagocytosis requires the full-length TREM2 receptor, SYK, PLCγ, and PI3K/AKT activities.

## Data Availability Statement

All datasets generated for this study are included in the manuscript/supplementary files.

## Author Contributions

HY, KC, JS, and DP performed the research. HY and DP designed the research and wrote the manuscript. HY, DP, MM, and FC analyzed the data. All the authors read and approved the final manuscript.

## Conflict of Interest

The authors declare that the research was conducted in the absence of any commercial or financial relationships that could be construed as a potential conflict of interest.
